# Supplementary figures and images for: One-step multiplex real-time RT-PCR assay for detecting and genotyping wild-type group A rotavirus strains and vaccine strains (Rotarix® and RotaTeq®) in stool samples
Source: PeerJ. 2016 Jan 11;4:e1560. doi: 10.7717/peerj.1560 (PMC4734446; doi:10.7717/peerj.1560)

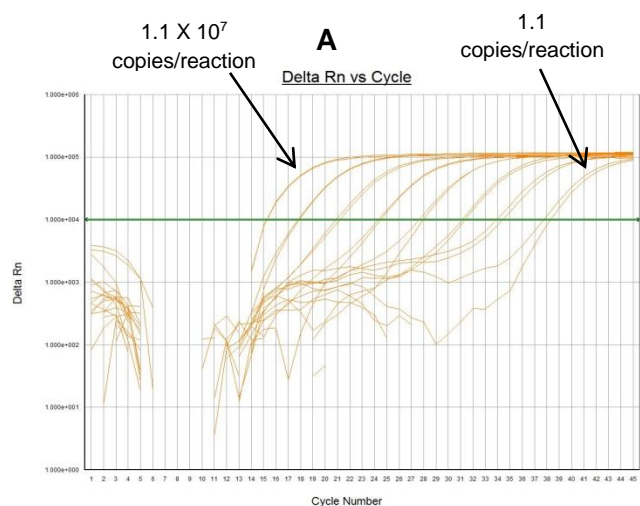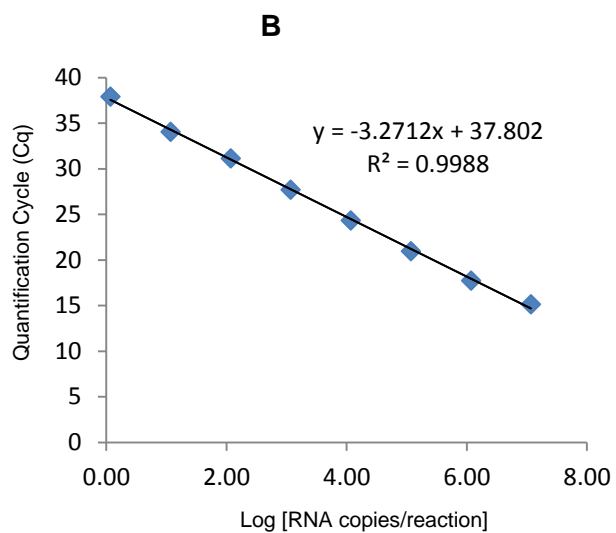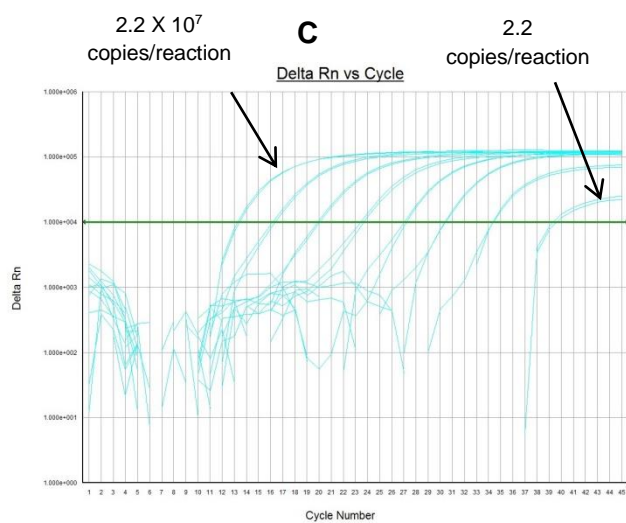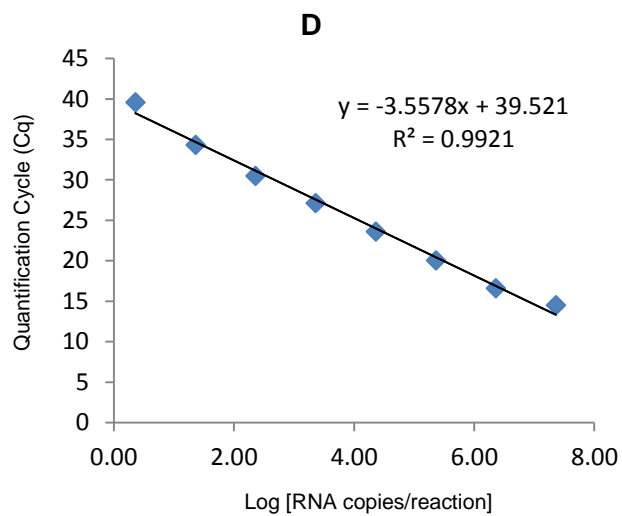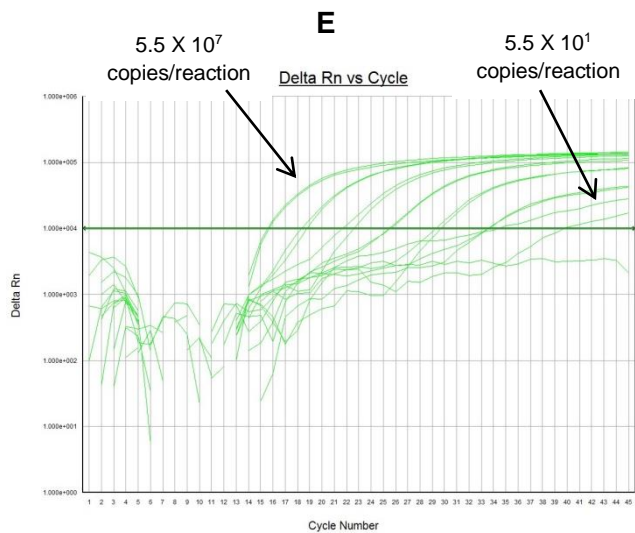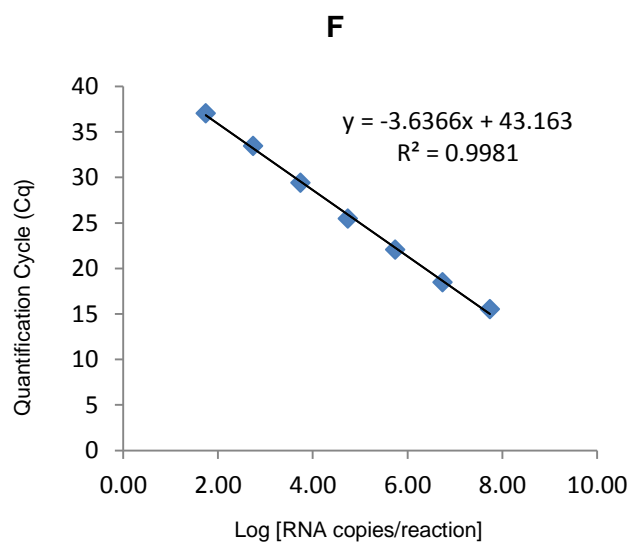

Supplement: Supplemental Information 1 — Amplification curves of (A) RotaTeq®-HEX, (C) Rotarix®-Cy5, (E) G12-FAM qRT-PCR using their respective 10-fold serial dilutions of dsRNA transcripts in singleplex reactions and the linear relationship between threshold cycle (Ct) and log transcript copy number per reaction (B) RotaTeq®-HEX, (D) Rotarix®-Cy5, (E) G12-FAM. Graphs showing the Ct value versus the log copy number were fitted with a regression line, and the slope for calculation of efficiency was obtained from the regression line. [file peerj-04-1560-s001.pdf]

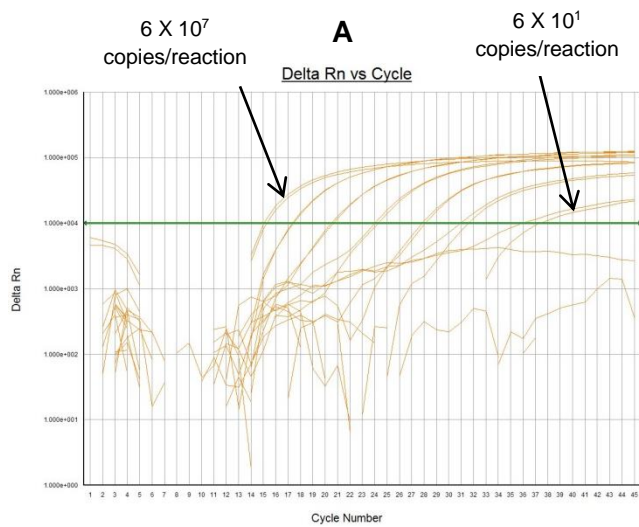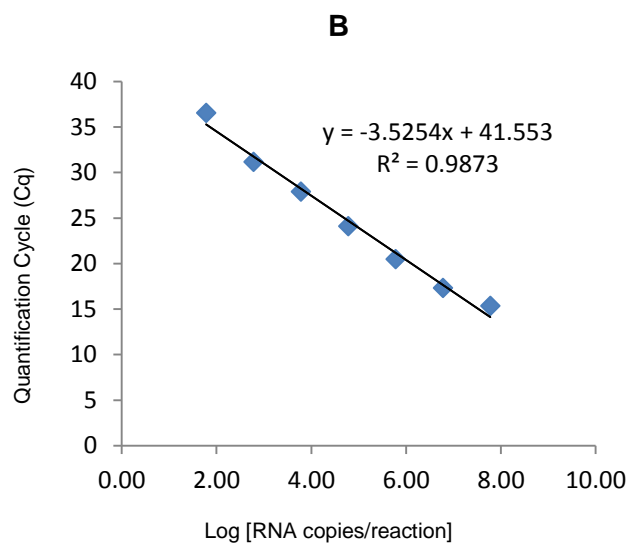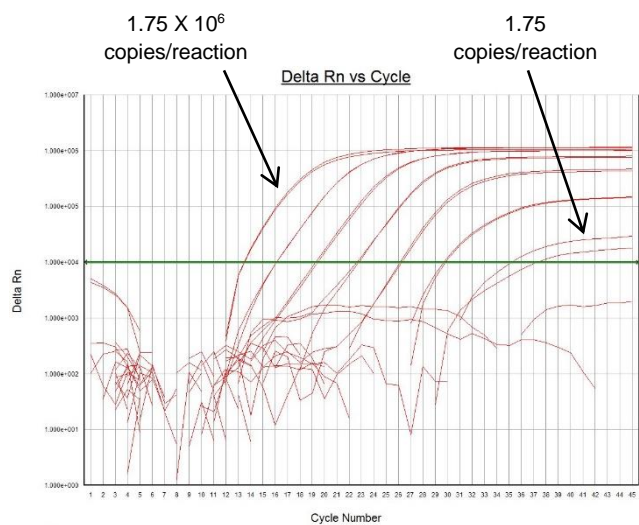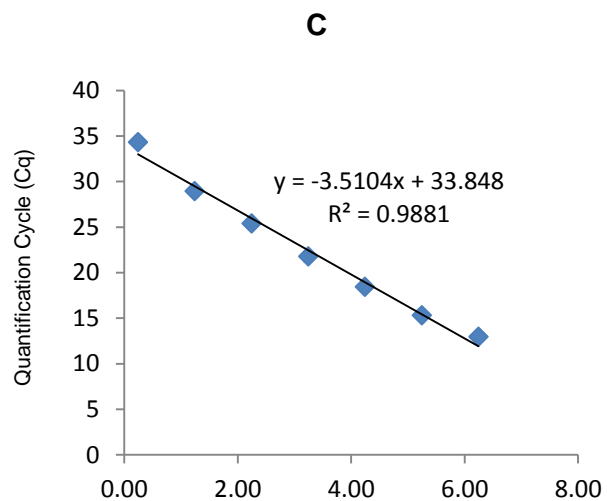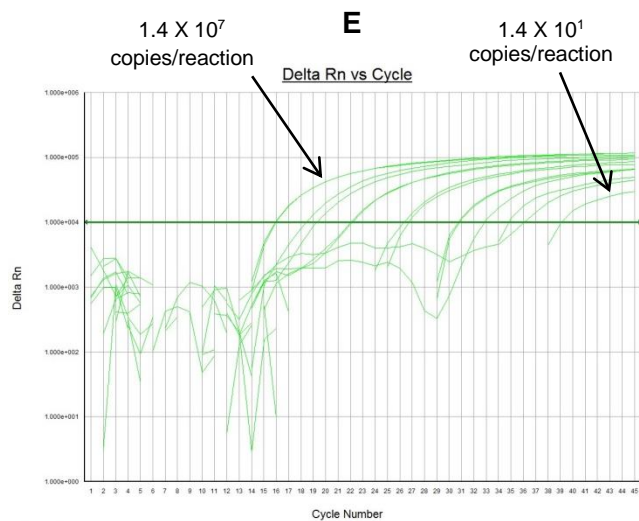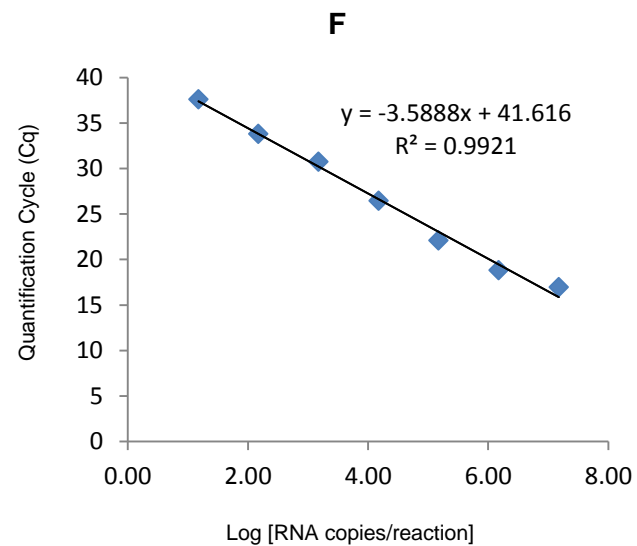

Supplement: Supplemental Information 2 — Amplification Curves of (A) G9-HEX, (C) NSP3-TR, (E) G4-FAM using their respective 10-fold serial dilutions of dsRNA transcripts in singleplex reactions and the linear relationship between threshold cycle (Ct) and log transcript copy number per reaction (B) G9-HEX, (D) NSP3-TR, (F) G4-FAM. Graphs showing the Ct value versus the log copy number were fitted with a regression line, and the slope for calculation of efficiency was obtained from the regression line. [file peerj-04-1560-s002.pdf]

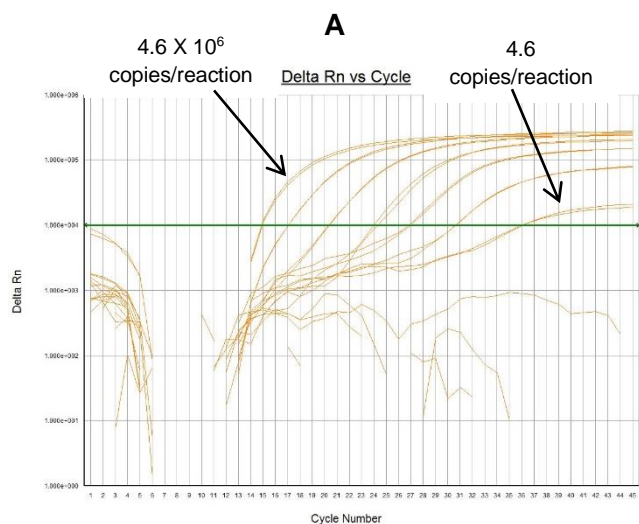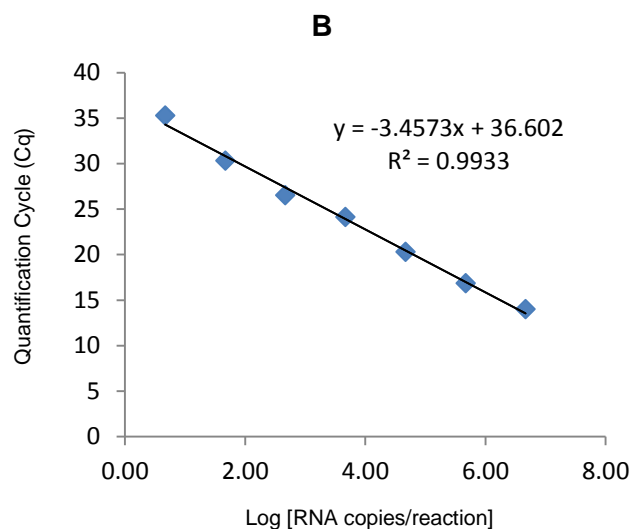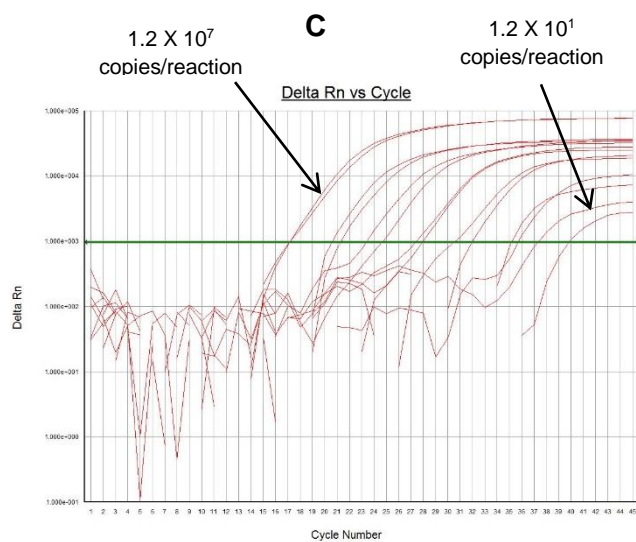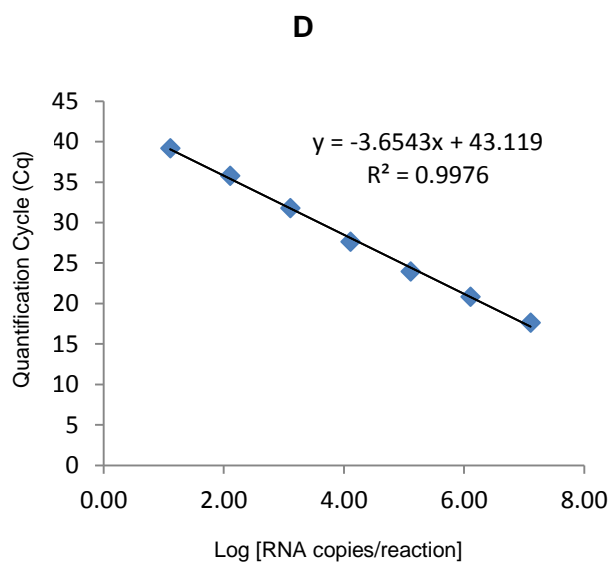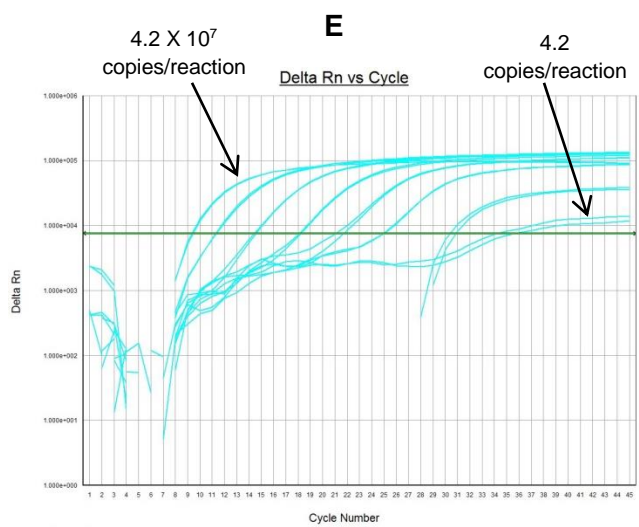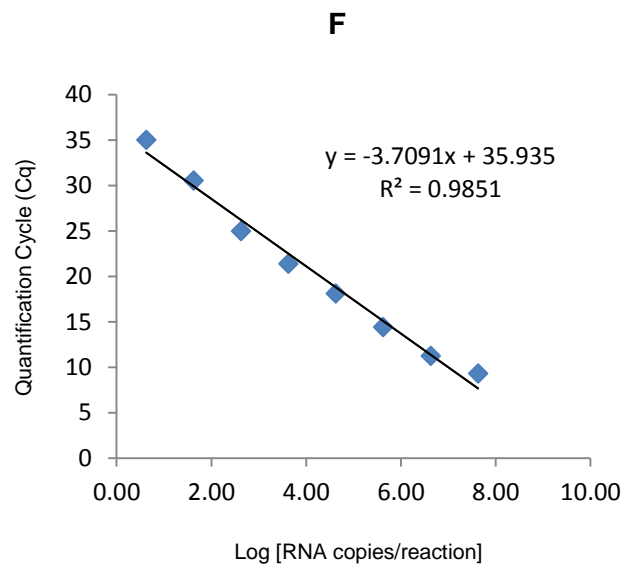

Supplement: Supplemental Information 3 — Amplification curves of (A) G1-HEX, (C) P[4]-TR, (E) G3-Cy5 using their respective 10-fold serial dilutions of dsRNA transcripts in singleplex reactions and the linear relationship between quantification cycle (Cq) and log transcript copy number per reaction (B) G1-HEX, (D) P[4]-TR, (F) G3-Cy5. Graphs showing the Cq value versus the log copy number were fitted with a regression line, and the slope for calculation of efficiency was obtained from the regression line. [file peerj-04-1560-s003.pdf]

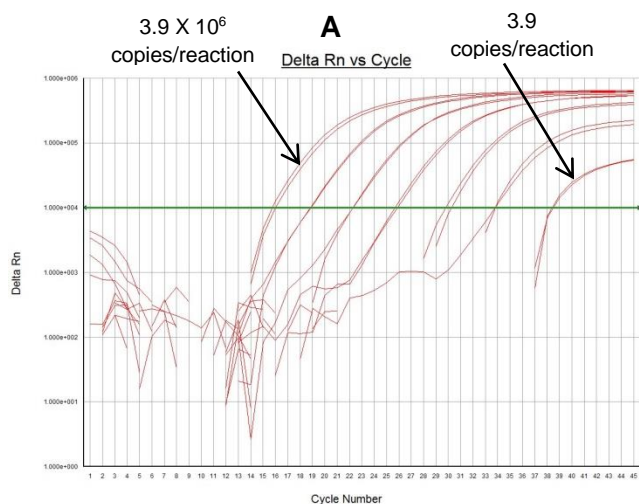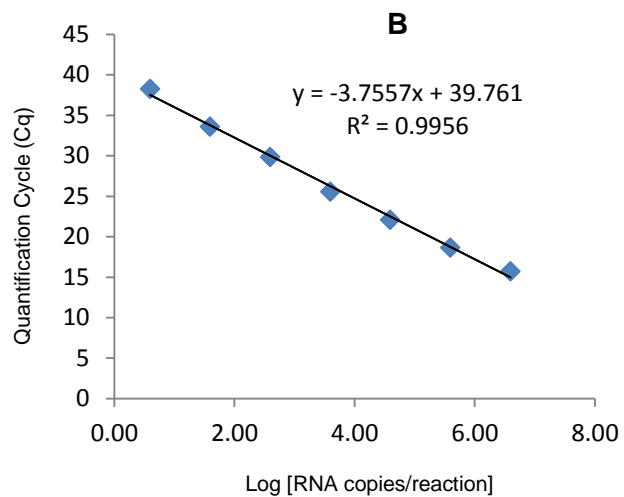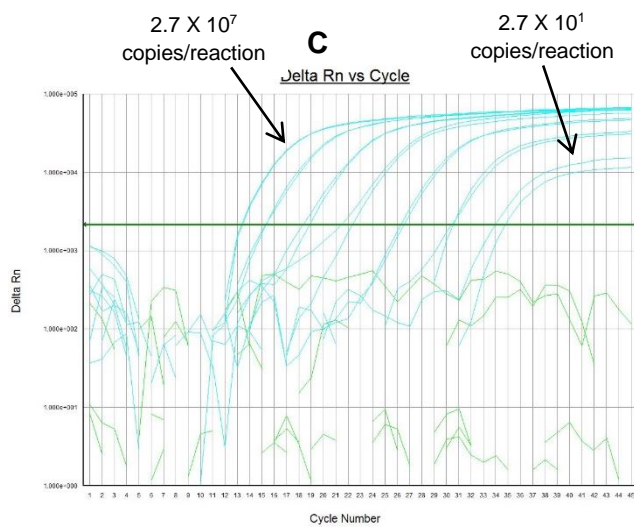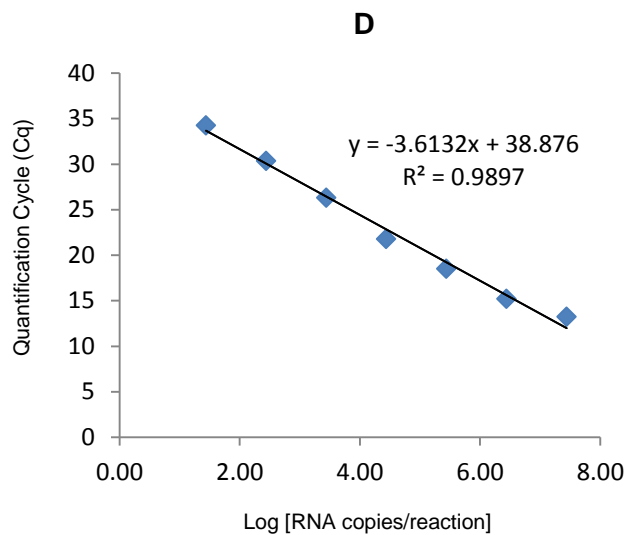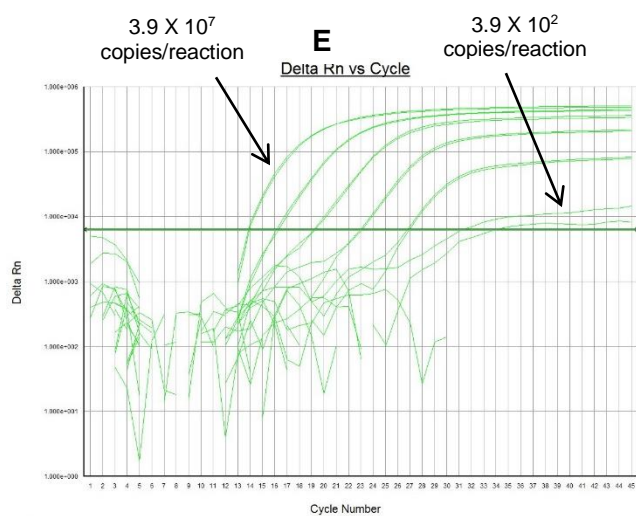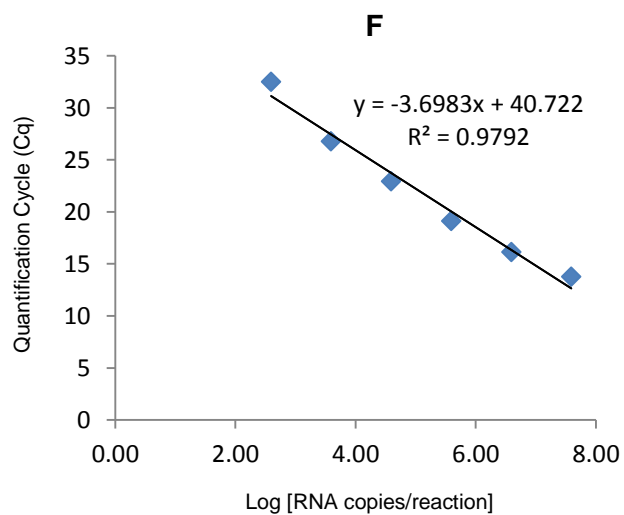

Supplement: Supplemental Information 4 — Amplification curves of (A) G2-TR, (C) P[8]-Cy5, (E) P[6]-FAM using their respective 10-fold serial dilutions of dsRNA transcripts in singleplex reactions and the linear relationship between threshold cycle (Ct) and log transcript copy number per reaction (B) G2-TR, (D) P[8]-Cy5, (F) P[6]-FAM. Graphs showing the Ct value versus the log copy number were fitted with a regression line, and the slope for calculation of efficiency was obtained from the regression line. [file peerj-04-1560-s004.pdf]
