# Supplementary material for: One-step multiplex real-time RT-PCR assay for detecting and genotyping wild-type group A rotavirus strains and vaccine strains (Rotarix® and RotaTeq®) in stool samples
Source: PeerJ. 2016 Jan 11;4:e1560. doi: 10.7717/peerj.1560 (PMC4734446; doi:10.7717/peerj.1560)
Supplement: Supplemental Information 5 — T7 promoter sequences shown in bold text. [file peerj-04-1560-s005.docx]

| **qRT-PCR assay** | **Primer name** | **T7 Promoter^a^ and Gene Specific Primer Sequences** | **Primer Position**  **(bp)** | **Tm**  **(°C)** | **Reference Strain**  **(accession #)** |
| --- | --- | --- | --- | --- | --- |
| **VP7** |  |  |  |  |  |
| VP7 G1 | T7VP7BEG9-F | **TAA TAC GAC TCA CTA TAG GGA GA**G GCT TTA AAA GAG AGA ATT TCC GTC TG | 1-27 | 67.4 | Wa (JX406755) |
|  | T7VP7END9-R | **TAA TAC GAC TCA CTA TAG GGA GAG** GTC ACA TCA TAC AAT TCT AAT CTA AG | 1061-1036 | 64.3 |  |
| VP7 G2 | T7VP7BEG9-F | **TAA TAC GAC TCA CTA TAG GGA GA**G GCT TTA AAA GAG AGA ATT TCC GTC TG | 1-27 | 67.4 | DS-1 (HQ650124) |
|  | T7VP7END9-R | **TAA TAC GAC TCA CTA TAG GGA GAG** GTC ACA TCA TAC AAT TCT AAT CTA AG | 1061-1036 | 64.3 |  |
| VP7 G3 | T7VP7BEG9-F | **TAA TAC GAC TCA CTA TAG GGA GA**G GCT TTA AAA GAG AGA ATT TCC GTC TG | 1-27 | 67.4 | P (AB118024) |
|  | T7VP7END9-R | **TAA TAC GAC TCA CTA TAG GGA GAG** GTC ACA TCA TAC AAT TCT AAT CTA AG | 1061-1036 | 64.3 |  |
| VP7 G4 | T7VP7BEG9-F | **TAA TAC GAC TCA CTA TAG GGA GA**G GCT TTA AAA GAG AGA ATT TCC GTC TG | 1-27 | 67.4 | ST3 (X13603) |
|  | T7VP7END9-R | **TAA TAC GAC TCA CTA TAG GGA GAG** GTC ACA TCA TAC AAT TCT AAT CTA AG | 1061-1036 | 64.3 |  |
| VP7 G9 | T7VP7BEG9-F | **TAA TAC GAC TCA CTA TAG GGA GA**G GCT TTA AAA GAG AGA ATT TCC GTC TG | 1-27 | 67.4 | WI61 (AB180969) |
|  | T7VP7END9-R | **TAA TAC GAC TCA CTA TAG GGA GAG** GTC ACA TCA TAC AAT TCT AAT CTA AG | 1061-1036 | 64.3 |  |
| VP7 G12 | T7VP7BEG9-F | **TAA TAC GAC TCA CTA TAG GGA GA**G GCT TTA AAA GAG AGA ATT TCC GTC TG | 1-27 | 67.4 | L26 (EF672595) |
|  | T7VP7END9-R | **TAA TAC GAC TCA CTA TAG GGA GAG** GTC ACA TCA TAC AAT TCT AAT CTA AG | 1061-1036 | 64.3 |  |
| **VP4** |  |  |  |  |  |
| VP4 P[4] | T7VP4-F | **TAA TAC GAC TCA CTA TAG GGA GA** GGC TAT AAA ATG GCT TC | 1-17 | 61.6 | DS-1 (AB118025) |
|  | T7VP4-R | **TAA TAC GAC TCA CTA TAG GGA GAG** GTC ACA TCC TCA ATA GCG TTC TC | 2359-2336 | 65.1 |  |
| VP4 P[6] | T7VP4-F | **TAA TAC GAC TCA CTA TAG GGA GA** GGC TAT AAA ATG GCT TC | 1-17 | 61.6 | ST3 (EF672612) |
|  | T7VP4-R | **TAA TAC GAC TCA CTA TAG GGA GAG** GTC ACA TCC TCA ATA GCG TTC TC | 2359-2336 | 65.1 |  |
| VP4 P[8] | T7VP4-F | **TAA TAC GAC TCA CTA TAG GGA GA** GGC TAT AAA ATG GCT TC | 1-17 | 61.6 | Wa (JX406750) |
|  | T7VP4-R | **TAA TAC GAC TCA CTA TAG GGA GAG** GTC ACA TCC TCA ATA GCG TTC TC | 2359-2336 | 65.1 |  |

**T7 promoter sequences shown in bold text**
